# The Global Spread of Hepatitis C Virus 1a and 1b: A Phylodynamic and Phylogeographic Analysis

**DOI:** 10.1371/journal.pmed.1000198

**Published:** 2009-12-15

**Authors:** Gkikas Magiorkinis, Emmanouil Magiorkinis, Dimitrios Paraskevis, Simon Y. W. Ho, Beth Shapiro, Oliver G. Pybus, Jean-Pierre Allain, Angelos Hatzakis

**Affiliations:** 1 Department of Hygiene, Epidemiology and Medical Statistics, Medical School, University of Athens, Athens, Greece; 2 Centre for Macroevolution and Macroecology, Research School of Biology, Australian National University, Canberra, Australia; 3 Department of Biology, The Pennsylvania State University, University Park, Pennsylvania, United States of America; 4 Department of Zoology, University of Oxford, Oxford, United Kingdom; 5 Department of Haematology, School of Clinical Medicine, University of Cambridge, Cambridge, United Kingdom; Massachusetts General Hospital (and Harvard Medical School), United States of America

## Abstract

**Background:**

Hepatitis C virus (HCV) is estimated to affect 130–180 million people worldwide. Although its origin is unknown, patterns of viral diversity suggest that HCV genotype 1 probably originated from West Africa. Previous attempts to estimate the spatiotemporal parameters of the virus, both globally and regionally, have suggested that epidemic HCV transmission began in 1900 and grew steadily until the late 1980s. However, epidemiological data suggest that the expansion of HCV may have occurred after the Second World War. The aim of our study was to elucidate the timescale and route of the global spread of HCV.

**Methods and Findings:**

We show that the rarely sequenced HCV region (E2P7NS2) is more informative for molecular epidemiology studies than the more commonly used NS5B region. We applied phylodynamic methods to a substantial set of new E2P7NS2 and NS5B sequences, together with all available global HCV sequences with information in both of these genomic regions, in order to estimate the timescale and nature of the global expansion of the most prevalent HCV subtypes, 1a and 1b. We showed that transmission of subtypes 1a and 1b “exploded” between 1940 and 1980, with the spread of 1b preceding that of 1a by at least 16 y (95% confidence interval 15–17). Phylogeographic analysis of all available NS5B sequences suggests that HCV subtypes 1a and 1b disseminated from the developed world to the developing countries.

**Conclusions:**

The evolutionary rate of HCV appears faster than previously suggested. The global spread of HCV coincided with the widespread use of transfused blood and blood products and with the expansion of intravenous drug use but slowed prior to the wide implementation of anti-HCV screening. Differences in the transmission routes associated with subtypes 1a and 1b provide an explanation of the relatively earlier expansion of 1b. Our data show that the most plausible route of the HCV dispersal was from developed countries to the developing world.

*Please see later in the article for the Editors' Summary*

## Abstract 2

Using phylodynamic and phylogeographic methods, Angelos Hatzakis and colleagues find that the global spread of Hepatitis C virus coincided with widespread use of transfused blood and with the expansion of intravenous drug use.

## Editors' Summary

**Background:**

About 150 million people (3% of the world's population) harbor long-term (chronic) infections with the hepatitis C virus (HCV) and about 3–4 million people become infected with this virus every year. HCV—a leading cause of chronic hepatitis (inflammation of the liver)—is spread through contact with infected blood. Transmission routes include medical procedures (for example, transfusions with unscreened blood) and needle-sharing among intravenous drug users. This second transmission route is the most common one in developed countries where blood is now routinely screened before being used in transfusions. HCV infection can cause a short-lived illness characterized by tiredness and jaundice (yellow skin and eyes), but most newly infected people progress to a symptom-free, chronic infection that can eventually cause liver cirrhosis (scarring) and liver cancer. HCV infections can be treated with a combination of two expensive drugs called interferon and ribavirin, but these drugs are ineffective in many patients.

**Why Was This Study Done?:**

Noone knows for sure where HCV originated although there is some evidence that it appeared first in West Africa or Southeast Asia. It is also unclear when the current HCV epidemic began. In this study, the researchers try to elucidate both the timescale and route of the global spread of the HCV epidemic by analyzing the genome sequence of HCV samples collected at different times and places. HCV is a ribonucleic acid (RNA) virus. That is, it stores the information it needs to replicate itself—its genome—as a series of “ribonucleotides.” Like other RNA viruses, the HCV genome continually accumulates small changes (mutations) and, over time, HCV has evolved into several different “genotypes,” each of which has several distinct subtypes. Furthermore, the viruses within a single subtype have subtly different genomes. By analyzing this viral diversity using complex “phylodynamic” and “phylogeographic” methods, scientists can build up a picture of how HCV has evolved in populations and how it has spread to reach its current geographical distribution.

**What Did the Researchers Do and Find?:**

By examining the genomes of HCV samples collected between 1994 and 2006 at the Athens University Medical School (Greece), the researchers first defined a variable region of HCV called E2P7NS2 that is more informative for phylodynamic studies than the NS5B region that has been used in previous studies. They then retrieved the sequences of both regions for subtype 1a and 1b samples collected over the past 20–30 years in the Los Alamos HCV sequence database; HCV subtypes 1a and 1b cause 60% of global HCV infections. The researchers' phylodynamic analyses of these globally representative sequences (collected in the USA, Germany, Switzerland, and Greece) indicate that the transmission of HCV subtype 1a occurred at a low rate from 1906 until the 1960s, at which time there was an explosive increase in its transmission rate. Similarly, subtype 1b transmission occurred at a low rate from 1922 until the late 1940s but then increased exponentially. From 1980 onwards, the prevalence of both subtypes stabilized at a high level. The researchers' phylogeographic analyses (which considered 1a and1b NS5B sequences collected in 21 and 29 countries, respectively) suggest that HCV subtypes 1a and 1b may have spread from the developed to the developing world.

**What Do These Findings Mean?:**

These findings indicate that the epidemic of HCV subtype 1b began in the 1940s when the use of transfused blood and blood products became widespread whereas the start of the subtype 1a epidemic coincided with the expansion of injected drug use that occurred in the 1960s. The findings also suggest that the transmission rates of both subtypes may have slowed before the widespread implementation of HCV screening in the early 1990s, possibly because the medical community was aware by then of the general risks associated with blood contamination. Finally, these findings provide new insights into how the HCV epidemic spread around the world and suggest that HCV may be evolving faster than previously thought. However, because this study relied on a small number of samples collected over a short time period, its findings need to be confirmed in larger studies.

**Additional Information:**

Please access these Web sites via the online version of this summary at http://dx.doi.org/10.1371/journal.pmed.1000198.

- The World Health Organization provides detailed information about hepatitis C and HCV
- The US Centers for Disease Control and Prevention provides information on hepatitis C for the public and for health professionals (information is also available in Spanish)
- The US National Institute of Diabetes and Digestive and Kidney Diseases provides basic information on hepatitis C (in English and Spanish)
- MedlinePlus provides links to further resources on hepatitis C
- The Los Alamos HCV database is available
- The US National Center for Biotechnology Information provides a science primer on how scientists reconstruct evolutionary pathways from sequence information

## Introduction

The World Health Organization (WHO) estimates that 3% of the world's population is infected by hepatitis C virus (HCV) [1]. HCV is primarily classified into six genotypes and many subtypes and, although its origin is unknown, patterns of viral diversity suggest an origin in either West Africa or Southeast Asia [2]–[4]. Even though the global HCV epidemic was widespread by 1980, it was not until 1989 that the virus was identified as the leading cause of non-A non-B hepatitis [5]. No animal source has been identified to support a hypothesis of zoonotic transmission [4].

The virus is transmitted by iatrogenic procedures and intravenous drug use (IDU) [1],[6]–[8]. Notably, several genotypes and subtypes have been associated with particular parenteral routes of transmission, for example 1b and 2 with transfusions, 1a and 3a with IDU [1], and 4a with unsafe injections in Egypt [9]. Infections with genotypes 1 and 4 are less responsive to interferon-based therapies than those with genotypes 2 and 3 [10]–[12].

Evolutionary (phylodynamic) analyses have been used successfully to infer aspects of the epidemic and transmission history of viruses such as dengue [13], HIV-1 [14]–[16], and influenza A [17]. This framework relies on the relationship between nucleotide sequence evolution and time, and has the ability to provide estimates of the infected population structure in the past [18]. Phylogeographic methods, which incorporate spatial information, have also been used to reconstruct the geographic dispersal of viruses such as HIV-1, HCV, and influenza A (Η5Ν1) [19]–[21] and are capable of describing the most plausible scenario of geographic expansion [22].

Previous attempts to estimate spatiotemporal dynamics of the global and regional spread of the HCV have suggested that epidemic transmission of HCV began around 1900 and expanded steadily until the late 1980s [20],[23]–[25]. However, the outcomes of these theoretical studies contrast with epidemiological evidence that the spread of HCV coincided with the massive increase of iatrogenic procedures and IDU around or after the mid-20th century [6].

In this study we aimed to elucidate the timescale and route of the global spread of HCV subtypes 1a and 1b by applying phylodynamic and phylogeographic methods.

## Methods

### Study Design

We first used a model dataset to identify and select the most phylogenetically informative HCV genome regions. Subsequently, we collated globally representative samples from the selected genome regions and applied an evolutionary analysis framework to infer the worldwide spatiotemporal dynamics of the HCV pandemic.

### The Model Dataset

A temporally stratified random sample (*n* = 97) of all available HCV 1a (*n* = 24), 1b (*n* = 27), 3a (*n* = 24), and 4a (*n* = 22) samples was selected from the serum bank of the Department of Hygiene, Epidemiology and Medical Statistics, Athens University Medical School (model dataset). These samples were obtained from different anonymized HCV-infected patients and collected during a 12-y period (1994–2006). One sample was selected per 6-mo period; when no sample was available in a specific 6-mo interval, the closest sample to that period was selected. In addition to the sampling date, the following information was recorded for each sample: patient's age, sex, ethnicity, transmission group, and treatment history. Samples were excluded if patients had a history of antiviral therapy and/or HIV co-infection, since these factors can affect the intrahost evolution of the virus [26]. Study approval was granted by the Institutional Review Board of Athens University Medical School. Epidemiological risk group information is summarised in Table S1.

### Selection of Genomic Region

**Figure 1:**
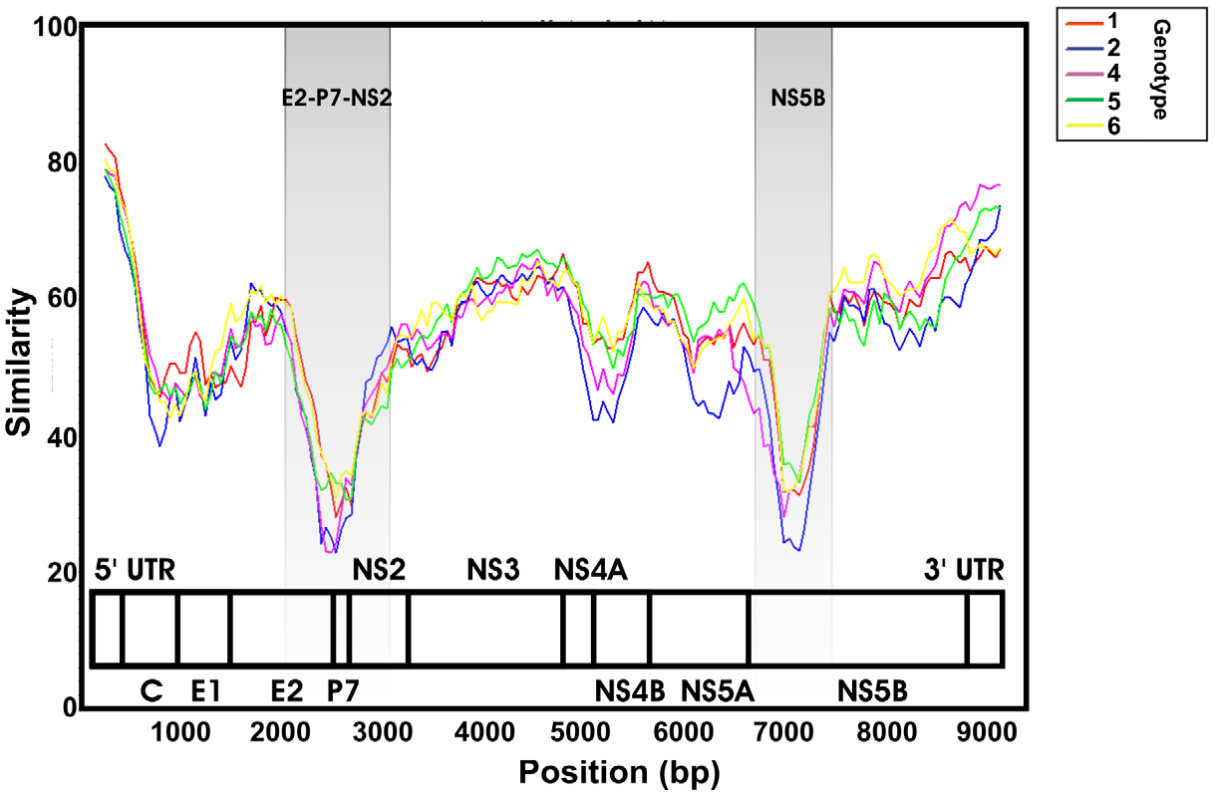
Similarity plot of the genotype 3 full-length reference sequence versus reference sequences for other genotypes along with the HCV genome. Similar plots are produced for all the genotypes. The shaded regions, E2P7NS2 and NS5B, are clearly the most divergent ones, and were selected for PCR-sequencing.

We constructed intergenotype similarity plots by means of the Simplot program [27] using a window of 500 nt, which was moved along the HCV genome in steps of 50 nt (Figure 1). These plots show that E2P7NS2 is the most divergent large (>450 nt) subgenomic region, followed by the 5′ end of NS5B. We therefore focused on sequencing regions E2P7NS2 and NS5B, thus enabling us to directly compare their molecular evolution, in the context of the molecular-clock assumption. We designed genotype-free primers for NS5B spanning nucleotides 8200–8800 (HCV-H reference strain numbering) and genotype-specific primers for E2P7NS2 spanning nucleotides 2540–3290. Sequencing was performed according to the manufacturer's instructions (3100 Avant Genetic Analyzer, Applied Biosystems). Primer sequences are listed in Table S2 and reverse transcription-PCR protocols are available upon request.

### The Global Dataset

In order to apply the optimized framework on a global scale, we retrieved all the available sequences for these two regions with known sampling dates up to September 2007 from the Los Alamos HCV sequence database (http://www.hcv.lanl.gov). Thus, we downloaded 87 and 86 sequences of NS5B and E2P7NS2, respectively, for genotype 1a, and 85 sequences of both NS5B and E2P7NS2 for genotype 1b (global dataset) (Table S3) [28],[29]. The sampling dates ranged from 1977 to 2007 for genotype 1a and from 1989 to 2006 for genotype 1b. The sampling locations were United States (*n* = 73), Switzerland (*n* = 11), and Germany (*n* = 3) for genotype 1a, and United States (*n* = 61), Switzerland (*n* = 22), Germany (*n* = 2), and Russia (*n* = 1) for genotype 1b. We also used the newly amplified subtype 1a and 1b Greek sequences. For genotypes 3a and 4a the availability of sequences with known sampling dates was insufficient (<10) to attempt a phylodynamic analysis.

### Power Optimization

We performed the analysis several times in order to find the most statistically powerful way to analyse our data: (i) each region (NS5B, E2P7NS2) was analysed separately; (ii) both regions were combined (concatenated) using strains for which sequences were available in both regions; (iii) the temporal information from both regions was combined, by applying the estimated E2P7NS2 time to most recent common ancestor (tMRCA) value as prior on the NS5B TMRCA for strains available in both regions. In the second case, we computed a combined likelihood as the product of partial likelihoods for each region. Each partial likelihood was computed using a distinct alignment and substitution model (GTR+gamma), but both partial likelihoods used the same tree (topology and scaled branch lengths).

### Demographic and Molecular Clock Model Selection

In order to select the best-fitting molecular clock and demographic model, we calculated the marginal likelihoods of the data conditional on all the evolutionary and demographic model parameters. We analysed all possible combinations of the relaxed [30] and strict molecular clock models and of the Bayesian skyline [18], constant, exponential, and logistic growth coalescent models. We excluded models that failed to converge or achieve sufficient chain mixing (effective sample size >100) before 30×10^6^ generations and after manual tuning of the sampler. As a result we estimated a Bayes Factors (BF) for each pair of models, as implemented in Tracer v1.4 and suggested previously [31].

### Phylogenetics and Phylodynamics

HCV genotype and subtype reference sequences were chosen as follows: Genotype 1 (1a: AF009606, AF387806, AF290978; 1b: D50483, AB049093, D85516); Genotype 2 (2a: AF169005, AB047645, D00944; 2b: AB030907; 2c: D50409); Genotype 3 (3a: D28917, D17763; 3b: D49374; 3k: D63821); Genotype 4 (Y11604); Genotype 5 (AF064490, Y13184); Genotype 6 (6a: Y12083; 6b: D84262; 6d:D84263; 6k: D84264; 6h: D84265; 6g: D63822).

Sequence alignment was performed using Clustal-W [32] and subsequently checked manually. We used ModelTest [33] to select the simplest evolutionary model that adequately fits the sequence data. Using PAUP [34], we estimated very large trees (>400 taxa) using Neighbor-Joining (under the Kimura 2-parameter substitution model) in order to determine the phylogenetic distribution of the included samples within the global epidemic. We estimated smaller trees using the program Tree-Puzzle [35] (under the Tamura-Nei substitution model [36]; rate heterogeneity among sites was modelled using a discrete gamma distribution with four rate categories). We used MEGA version 4 [37] to visualize and decorate the constructed trees. We used root-to-tip regression (as implemented in the program Path-o-gen; http://tree.bio.ed.ac.uk; [38]) as an exploratory tool to evaluate the clock-likeness of the sequenced regions.

We performed phylodynamic analysis using the framework implemented in BEAST [39]. Markov Chains Monte Carlo (MCMC) sampling was performed for at least 1×10^7^ generations, sampling a tree every 1000 generations. We used the General Time Reversible model of nucleotide substitution, with rate heterogeneity among sites modelled using a discrete gamma distribution with four rate categories. The program Tracer (http://tree.bio.ed.ac.uk) was used to check for convergence and to determine whether appropriate mixing of the posterior target distribution had been achieved (effective sample size >100).

We fitted a shifted bivariate gamma distribution to the posterior distribution of each tMRCA parameter. This was achieved by maximum likelihood using the gammafit function implemented in STATA 8.0 [40]. We calculated the shift of the gamma distribution as being equal to the modulus of the minimal value of the estimated tMRCA distribution.

### Phylogeography

To track the historical spread of HCV 1a and 1b epidemics, we reconstructed viral dispersal by applying Slatkin and Maddison's phylogenetic method for inferring migratory events [41] to all available 1a and 1b NS5B sequences by means of the Mesquite program [42]. This method has previously been used to estimate viral dispersal of the influenza A (H5N1), HIV, and HCV epidemics [19]–[21]. Because our sample is not representative of the country-specific epidemics, we are not interested here in the quantitative features of the phylogeographic analysis (migration matrix). Instead, we intend to simply describe qualitative aspects of the phylogeography such as the degree of geographic dispersal and the most plausible origin of the current sample. This approach was chosen because the global trees were not conclusive about the origin of the HCV 1a and 1b subepidemics in different countries, owing to the absence of a monophyletic country-specific outlier. Both HCV 1a and 1b trees were rooted using the other subtype, i.e., to root subtype 1a we used all the available subtype 1b strains and vice versa. The phylogeographic analysis was performed independently for each subtype without taking into account the outgroup.

For this analysis we classified as developed countries: Australia, Belgium, Canada, France, Germany, Great Britain, Greece, Ireland, Japan, Spain, Switzerland, and the United States. We classified as developing countries: Argentina, Brazil, Cameroon, China, Egypt, India, Iran, Korea, Martinique, Mongolia, Nepal, Peru, Philippines, Russia, Singapore, Taiwan, Thailand, Tunisia, Turkey, Uzbekistan, and Vietnam

### Accession Numbers

Subtype 1a and 1b sequence accession numbers from the model dataset have been deposited in GenBank (http://www.ncbi.nlm.nih.gov/Genbank) with the following accession numbers: FJ538017–FJ538098

## Results

### The Model Dataset

In order to obtain the best molecular clock signal we amplified and sequenced two specific regions of HCV 1a, 1b, 3a, and 4a, the NS5B (nt 8200–8800 [nucleotide position in relation with the HCV-H reference strain]) and the E2P7NS2 (nt 2540–3290) regions (see Figure 1). For the model dataset comparison of the NS5B and E2P7NS2 regions shows that E2P7NS2 outperforms the NS5B in terms of evolutionary linearity or “clocklike-ness.” This finding is apparent in regressions of genetic distance against sampling time (Figure S1) and in the results of relaxed molecular clock analyses (Table 1). Thus a strict clock model adequately fits E2P7NS2, whereas NS5B should be modelled by a relaxed-clock model (Table 1) [30],[43]. The coalescent population parameters were similar for both regions, suggesting that NS5B retains substantial information about the shape of genealogy of the strains (unpublished data). The E2P7NS2 region also outperforms NS5B in terms of the precision of tMRCA, showing that the co-estimation of evolutionary rates and the tMRCA in a single step is feasible (here, “precision” is used in its statistical sense, i.e., the inverse of the estimation variance). In addition, using the time-scale estimated from E2P7NS2 as a prior during the analysis of NS5B resulted in more precise estimates of the tMRCA and population dynamics (data available on request). These results suggest that E2P7NS2 offers a significant improvement for estimating the epidemic history of HCV subtypes.

**Table 1:** Comparison of estimated coefficient of variation parameter for each subtype and genome region.

Subtype	E2P7NS2	NS5B
Subtype 1a	0.092 (0.00002–0.274)	0.31 (0.02–0.56)
Subtype 1b	0.108 (0.00004–0.233)	0.26 (0.02–0.44)
Subtype 3a	0.14 (0.00005–0.334)	0.44 (0.07–0.74)
Subtype 4a	0.117 (0.00007–0.36)	0.48 (0.15–0.84)

Under a relaxed molecular clock, this value represents the clocklike-ness of sequence evolution (lower values represent less among-branch variation in evolutionary rate). The 95% highest posterior density (HPD) intervals of each estimate are given in parentheses.

### The Global Dataset

**Figure 2:**
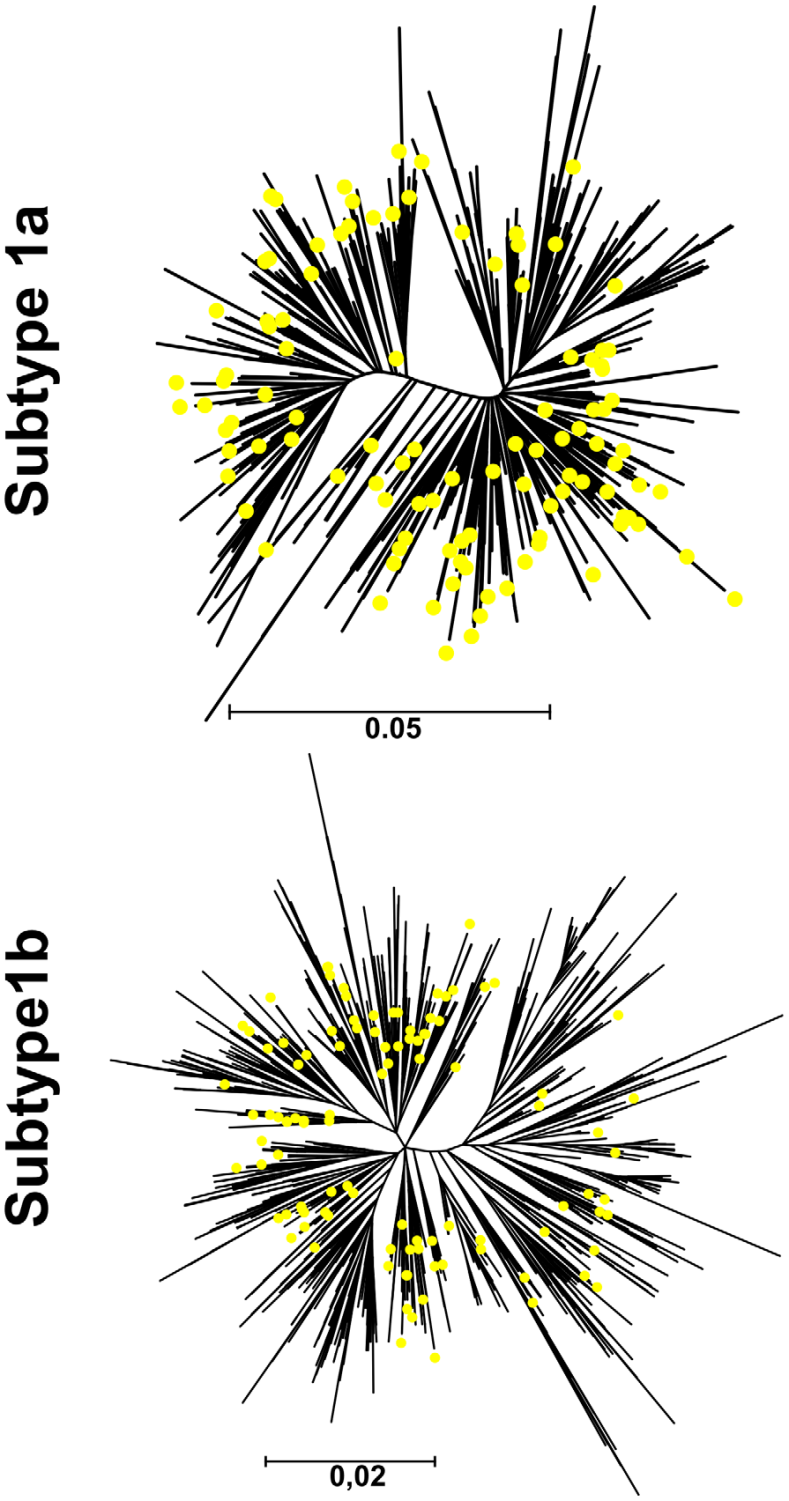
Phylogenetic trees of the isolates used in the population dynamics (yellow circles) along with all the available NS5B sequences (tips without circles).

To determine whether the global datasets used in the phylodynamic analysis were representative of the global HCV epidemic, we downloaded all available 1a and 1b sequences for a smaller part of NS5B (nucleotides 8297–8597), for which a much greater number of sequences was available (992 sequences from 21 countries for subtype 1a; 1,208 sequences from 29 countries for subtype 1b; details in Table S4). The same dataset (alignment available upon request) was used in the phylogeographic analysis (see below). The phylogenetic trees estimated from the smaller NS5B (nt 8297–8597) region indicate that the global dataset is representative of the global epidemic (Figure 2). Thus the tMRCA of the global dataset sequences is a fair approximation of the tMRCA of the global epidemic, allowing the results from a sample to be projected and generalized to the global epidemic as a whole, as has been reported previously for HIV [14]. A detailed investigation into random sampling of the dated sample from the globally available sequences is presented in the first part of Text S1.

We performed a phylodynamic analysis using the program BEAST [39]. In order to select the best fitting model we investigated parametric and nonparametric models for population growth [18] and strict and relaxed-clock models [30] of molecular evolution. We found that in each case (subtype 1a or 1b, region E2P7NS2 or NS5B) the best fitting model was the relaxed molecular clock model plus the Bayesian skyline demographic model (Tables 2, S5, and S6). Interestingly, for subtype 1a the evidence against a strict clock in the E2P7NS2 region was weak, indicating that this region is more consistent with a strict clock than NS5B. As a result we chose the best fitting Bayesian skyline and relaxed-clock models and thus made no parametric assumptions about demographic history. We also found that the precision of tMRCA was maximized (Tables 2 and S7) when the estimated tMRCA of the E2P7NS2 region was used as a prior on the tMRCA of the less informative NS5B region.

**Table 2:** Estimated timescale of the global dataset using a relaxed-clock model.

Genomic Region	Date of MRCA	Rate (10^−3^ substitution/site/y)	CoV
Subtype 1a			
E2-P7-NS2	1914 (1818–1956)	1.3 (0.055–2,1)	0.20
NS5B	1900 (1802–1957)	1.0 (0.7–1.4)	0.25
NS5B with E2-P7-NS2 prior	1931 (1906–1957)	1.0 (0.72–1.4)	0.25
Subtype 1b			
E2-P7-NS2	1944 (1905–1965)	2.1 (1.1–3.0)	0.230
NS5B	1911 (1806–1959)	1.2 (0.42–2.0)	0.32
NS5B with E2-P7-NS2 prior	1940 (1922–1963)	1.9 (1.2–2.6)	0.32

The 95% highest posterior density (HPD) intervals of each parameter are given in parentheses. CoV, coefficient of variation.

### Evolutionary Rates

Interestingly, previously reported estimates of the substitution rate for HCV NS5B (5×10^−4^ substitutions/site/year) were close to the lower bound of our NS5B rate credibility interval (Table 2). When the E2P7NS2 tMRCA was used as a prior on the NS5B tMRCA, the estimated rate for NS5B (1–1.9×10^−3^ substitutions/site/year) is about 2–4 times faster than previously estimated [25],[44]. However, previous analyses used a smaller part of NS5B, making it difficult to compare estimated rates. In order to directly compare these estimates we truncated our subtype 1b NS5B alignment to match the region used in the previous studies [44] and repeated the analysis (previous estimates of comparable subtype 1a rates were not available). We estimated the rate of this truncated region to be 2.5×10^−3^ substitution/site/year (95% highest posterior density 1.5–3.7×10^−3^), which again is faster than previously estimated.

### The Temporal Spread of HCV 1a and 1b

**Figure 3:**
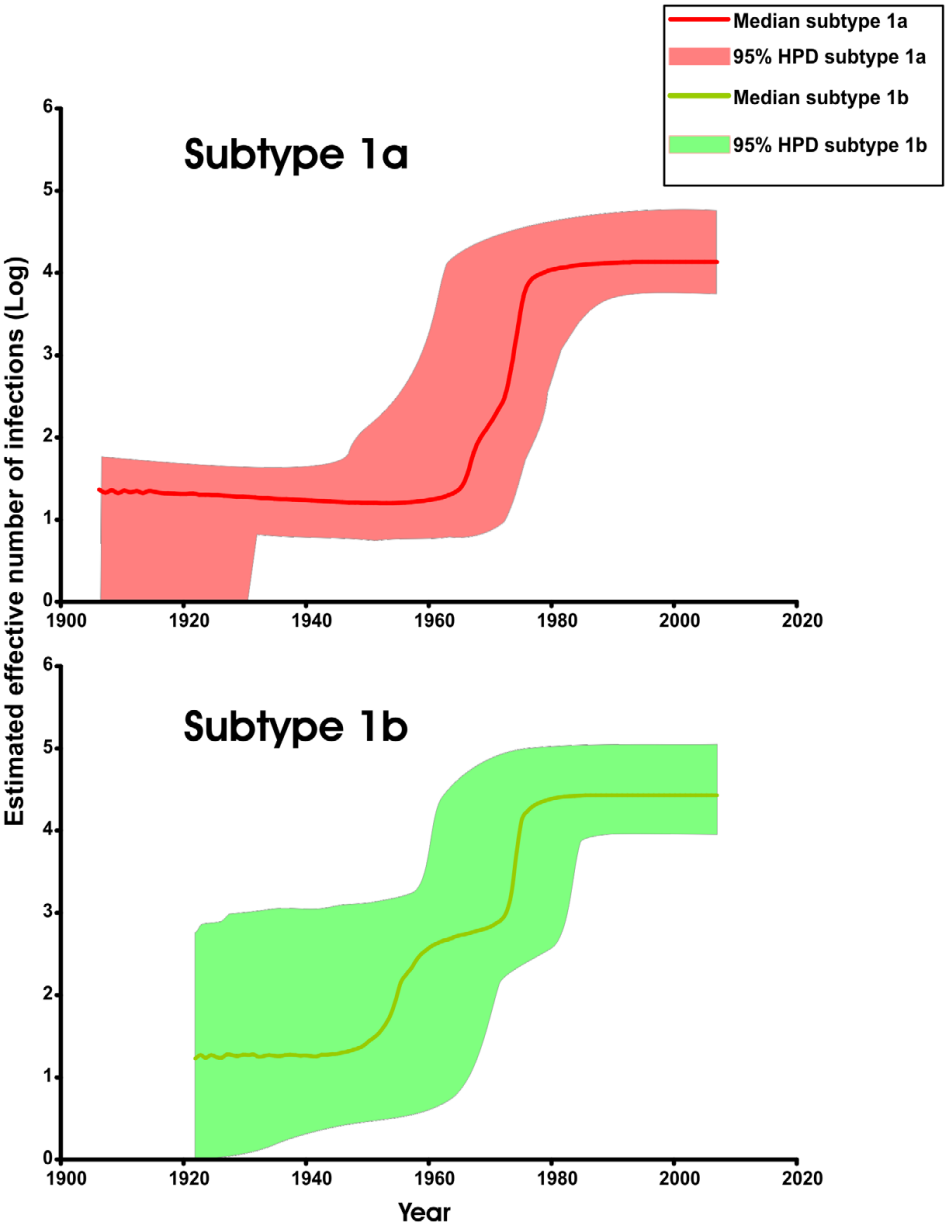
Global population dynamics of the hepatitis C virus genotypes 1a and 1b based on relaxed-clock analysis of NS5B. The tMRCA estimated from E2P7NS2 was used to provide a gamma-distributed prior for the tMRCA of strains also available for NS5B.

The Bayesian skyline plot summarizes the spread and epidemic growth of the globally prevalent HCV genotypes 1a and 1b (Figure 3). It clearly shows that subtype 1a was in a steady nonexpanding phase maximum from around 1906 (the lower 95% credible interval of the tMRCA) to the 1960s, after which it expanded explosively until around 1980. The subtype 1b epidemic was in a steady nonexpanding phase maximum from 1922 (the lower 95% credible interval of the tMRCA) to the late 1940s. Subsequently the subtypes grew exponentially up to the 1980s. Similar results were obtained when we excluded the newly amplified and sequenced 1a and 1b Greek strains from the analysis (data available upon request). The spread of subtype 1b preceded that of subtype 1a by approximately 16 y (95% confidence interval [CI] 15–17) (Text S1).

### HCV 1a and 1b Phylogeography

**Figure 4:**
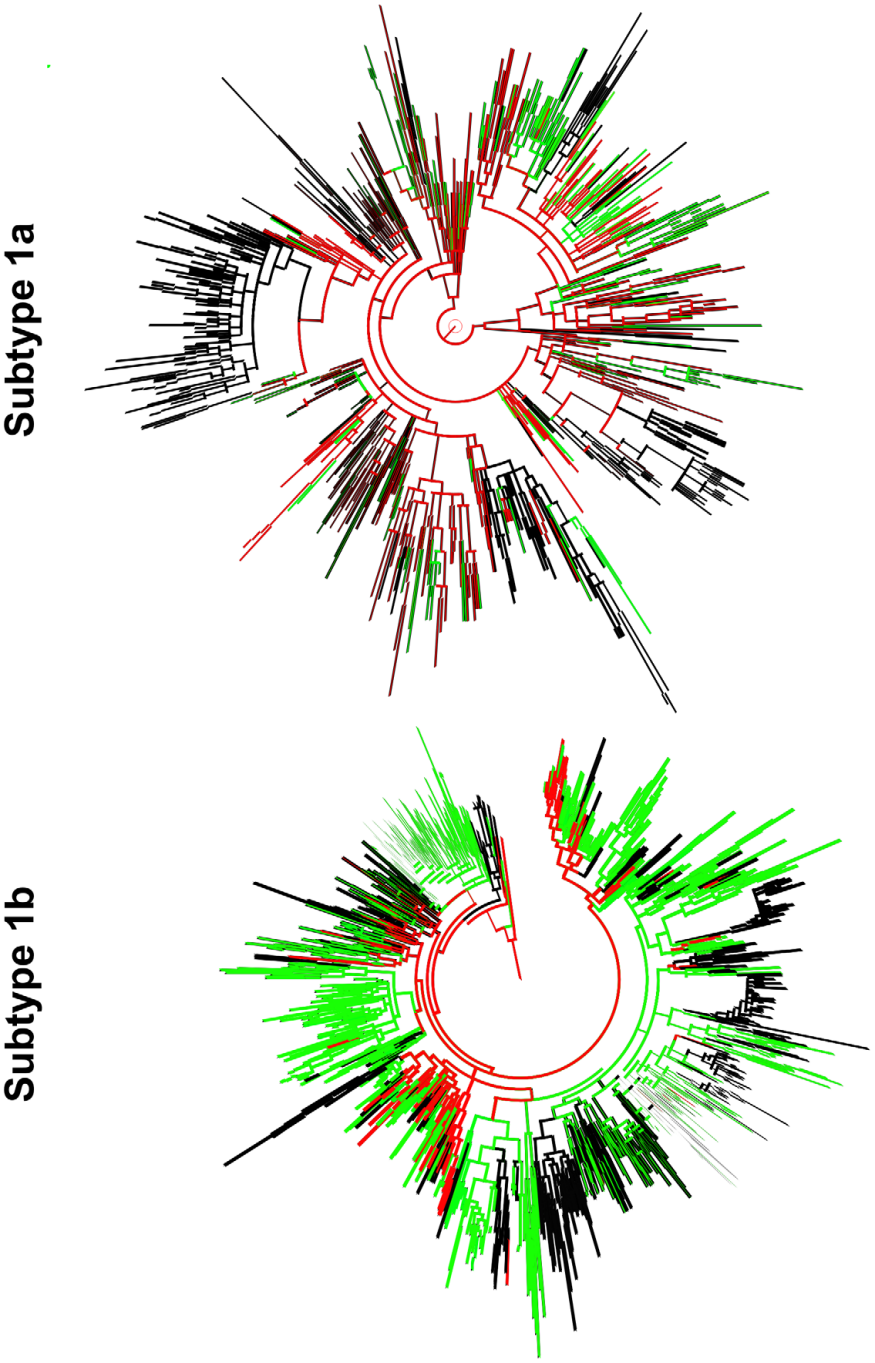
Phylogeographic trees of all the partial NS5B sequences available for 1a and 1b subtypes. The red, green, and black lines indicate events attributed to the sequences sampled from the US, other developed, and developing countries, respectively.

Generally, the hierarchy presented in both phylogeographic trees (subtype 1a and 1b) (Figure 4) suggests that the earliest divergence events occurred in developed countries, whilst spread to developing countries tends to be limited to the most recent terminal parts of the tree. In order to further investigate the most plausible source of the global 1a and 1b epidemic we constructed nonclock phylogenetic trees and annotated them with country-specific monophyletic clusters and estimates of cluster dates of origin (where available; see Figure S2). Both trees indicate that strains from developed countries are dispersed across the whole tree either as independent lineages or as outliers within cluster of strains from developing countries; this dispersion is estimated to have occurred in a period of 10 y for both subtypes (Figures S2 and S3).

## Discussion

Our analysis aimed to estimate the spatiotemporal spread of the global epidemic HCV subtypes 1a and 1b. First, we were able to improve significantly the analytical framework of HCV phylodynamics by demonstrating that E2P7NS2 is evolving in a more clocklike manner than NS5B. Moreover, we showed that HCV is evolving faster than previously thought and we were able to provide more precise estimates of the timescale and dynamics of epidemic growth for subtypes 1a and 1b. These estimates support a massive expansion of the epidemics between 1940 and 1980, as opposed to the previous conjecture of a more continual and steady increase across the whole of the 20th century.

This time frame suggests that the global epidemic of both subtypes was possibly initiated and sustained by the vast increase of parenteral iatrogenic procedures during and after World War II (transfusions, plasma pooling, and unsafe therapeutic injections) [43]–[45]. Blood and freeze-dried (lyophilized) pooled plasma could have served as a vehicle for global HCV dissemination, for the following reasons: (i) pooling of plasma increased the possibility of containing and transmitting the virus; (ii) freeze-dried plasma could be stored easily for a long period and used far from the blood donation site; (iii) the dry plasma remains infectious; and (iv) there is ample historical evidence for the shipment of plasma and stored red cells around the world [45]. The high frequency of subclinical primary HCV infection and nonspecific symptoms of other cases might have permitted such an outbreak to escape attention.

The observed epidemic growth also coincides with the history of illicit IDU; the US and Canada have the longest history of IDU, which developed in the late 1920s and spread in the 1930s [46]. Before the Second World War about 40% of addicts seeking treatment were injecting, and this figure had risen to 70%–90% by 1950 [46],[47]. A peak of heroin use in North America occurred at end of the 1960s [48]; injecting heroin was especially common in military servicemen, veterans [49], and inner city populations [48]. In Europe and Australia the spread of IDU began in the late 1960s [46]. In Asia the first important IDU epidemic (amphetamine use) was in Japan between 1946 and 1956, while in Hong Kong heroin injecting has been documented since the 1950s [46]. Interestingly, IDU is relatively recent in many Asian countries such as China, India, Lao People's Democratic Republic, Myanmar, Nepal, Sri Lanka, and Vietnam [46].

The expansion of HCV subtype 1b preceded that of subtype 1a by at least 16 y (95% CI 15–17), and it probably coincides with the vast increase in transfusions and unsafe therapeutic injections, whereas the expansion of HCV 1a is more strongly associated with the increase in IDU after 1960. Our analysis suggests that the exponential expansion of HCV 1a and 1b reached a plateau in the 1980s, possibly prior to implementation of anti-HCV screening at the beginning of the 1990s. These results are consistent with epidemiological data indicating that the incidence of acute non-A, non-B hepatitis, and HCV infection greatly increased from the 1960s to the early 1980s and declined before 1990 in the US, Italy, France, and Greece [50]–[54]. This decline was probably due to increased awareness of the medical community to parenteral risks, better blood donor selection, HBsAg, ALT, anti-HBc, anti-HIV screening, and the use of viral inactivation of clotting factor concentrates [55].

Our findings are also corroborated by: (i) HCV data from US military recruits, which indicate that all genotyped samples collected during 1948–1955 were found to be subtype 1b [56], (ii) by modelling data on the incidence of HCV infection in the US haemophilia population, which is mainly infected with HCV 1a [57], and (iii) by demography of local epidemics where HCV 1b infected individuals are systematically older than HCV 1a infected ones [2],[58]. Moreover, the rise in subtypes 1a and 1b also coincided with the rise of syringe availability [6] and the trends of IDU in the US [48] and globally [46].

Our phylogeographic analysis indicated that HCV subtypes 1a and 1b most probably expanded from the developed countries to the developing world. However, our approach is not immune from sampling bias since many countries have been under-represented and a few over-represented (e.g., 399 1a sequences and 89 1b sequences from the US, 129 1b sequences from China, 276 1b sequences from Japan, and 276 1b sequences from Spain; Table S4); in addition most of the sequences do not have sampling date information. As a result we were not able to perform a full quantitative analysis of the rates and modes of HCV transmission among countries, but instead can only describe the qualitative aspects of the phylogeographic tree (such as geographic dispersal and origin), which are likely to be more robust to sampling bias. The observed phylogeographic patterns of both subtypes are similar and can be described as a source-sink pattern, with developed countries representing the source of the spatial spread of the epidemic [22]. These descriptive patterns of the estimated phylogeography suggest that the first wave of transmission (probably from plasma and blood transfusions) facilitated the spread of HCV initially to developed countries and subsequently to the developing world where local epidemics were further established from location specific iatrogenic procedures and IDU [46].

Interestingly, subtype 1b has been found to be predominant in all countries with a high prevalence of hepatocellular carcinoma (HCC), including Japan, Italy, and Spain [59]. Since our analysis suggests that 1b preceded the 1a epidemic by ∼16 y, this association can be explained by the epidemic being older in these countries. This finding is reinforced by the observation that the prevalence of subtype 1b infection in HCC patients is higher than that of subtype 1a [60],[61]; although a higher oncogenic activity of 1b cannot yet be entirely excluded, one other plausible explanation is that 1b infections are older and thus more likely to develop severe liver disease. If this is the case then our analysis predicts that the seroepidemiology of HCV in liver disease patients will eventually change and that the relative incidence of 1a HCC cases will increase.

This analysis provides a framework for applying established phylodynamic methods to the estimation of HCV epidemic spread, by using the NS5B and E2P7NS2 genomic regions, the latter being only rarely sequenced. Although data suggest that HCV genotype 1 as whole is endemic in West Africa and thus may have originated there [2],[3], we show that the most prevalent HCV subtypes 1a and 1b expanded globally after World War II, probably through widespread availability of blood transfusions and blood products, invasive medical procedures, use of unsafe therapeutic injections, and widespread use of IDU.

## Supporting Information

Figure S1 Regression of root-to-tip genetic distances against sampling date for the E2P7NS2 and NS5B regions in the model dataset (genotype 3a). The root has been chosen as the branch that maximizes the coefficient of determination (Pearson's *r*), under the assumption of a strict molecular clock.
(0.41 MB TIF)

Figure S2 Phylogenetic trees of all partial NS5B sequences available for subtypes 1a (also presented as phylogeographic trees in Figure 2). This figure shows the phylogenetic trees annotated with dated nodes (median dates, blue numbers) and country-specific clusters (colored triangles). Each country-specific cluster is comprised of at least four taxa and contains at least 80% strains isolated from the specified country. Country codes are: ES (Spain), TN (Tunisia), US (United States of America), FR (France), GB (Great Britain), CH (Switzerland), BR (Brazil), PH (Philippines), TW (Taiwan), IE (Ireland), RU (Russia), IN (India), JP (Japan), CN (China), MN (Mongolia), VN (Vietnam). Red circles indicate dispersed strains isolated from the US.
(0.40 MB TIF)

Figure S3 Phylogenetic trees of all partial NS5B sequences available for subtypes 1b (also presented as phylogeographic trees in Figure 2). This figure shows the phylogenetic trees annotated with dated nodes (median dates, blue numbers) and country-specific clusters (colored triangles). Each country-specific cluster is comprised of at least four taxa and contains at least 80% strains isolated from the specified country. Country codes are: ES (Spain), TN (Tunisia), US (United States of America), FR (France), GB (Great Britain), CH (Switzerland), BR (Brazil), PH (Philippines), TW (Taiwan), IE (Ireland), RU (Russia), IN (India), JP (Japan), CN (China), MN (Mongolia), VN (Vietnam). Red circles indicate dispersed strains isolated from the US.
(0.42 MB TIF)

Table S1 Epidemiological risk group distribution for each HCV subtype in the model dataset. Description of primers used in the experimental phase.
(0.03 MB DOC)

Table S2 Primers used to amplify the sequences forming the model dataset. For genotypes 1a, 1b, and 4a we have implemented a semi-nested approach for the E2P7NS2 region.
(0.04 MB DOC)

Table S3 Sequences of the global dataset, together with their spatiotemporal sampling information.
(0.20 MB DOC)

Table S4 Country distribution of the global dataset.
(0.02 MB PDF)

Table S5 Model selection results for the subtype 1a global dataset. ln-likelihoods and log10 Bayes factors (BF) for each pair of models (model 1 = row versus model 2 = column). A log10 BF>5 (decibans) is substantial evidence and >10 is strong evidence for the support of model 1 over model 2.
(0.05 MB DOC)

Table S6 Model selection results for the subtype 1b global dataset. ln-likelihoods and log10 Bayes factors (BF) for each pair of models (model 1 = row versus model 2 = column). A log10 BF>5 (decibans) is substantial evidence and >10 is strong evidence for the support of model 1 over model 2.
(0.04 MB DOC)

Table S7 Comparison of the precision of different data combinations in estimating the tMRCA in the global dataset (95% higher posterior probability). It is easily shown that maximum precision is achieved when E2P7NS2's estimate of the tMRCA is applied as a prior on the tMRCA of NS5B.
(0.03 MB DOC)

Text S1 Testing for random sampling and statistics about the time lag between subtype 1a and 1b epidemics. Analysis of Figures S1, S2, and S3.
(0.04 MB DOC)

## Abbreviations

CI: confidence interval
HCV: hepatitis C virus
IDU: intravenous drug use
tMRCA: time to most recent common ancestor
HCC: hepatocellular carcinoma
