# Supplementary figures and images for: The Global Spread of Hepatitis C Virus 1a and 1b: A Phylodynamic and Phylogeographic Analysis
Source: PLoS Med. 2009 Dec 15;6(12):e1000198. doi: 10.1371/journal.pmed.1000198 (PMC2795363; doi:10.1371/journal.pmed.1000198)

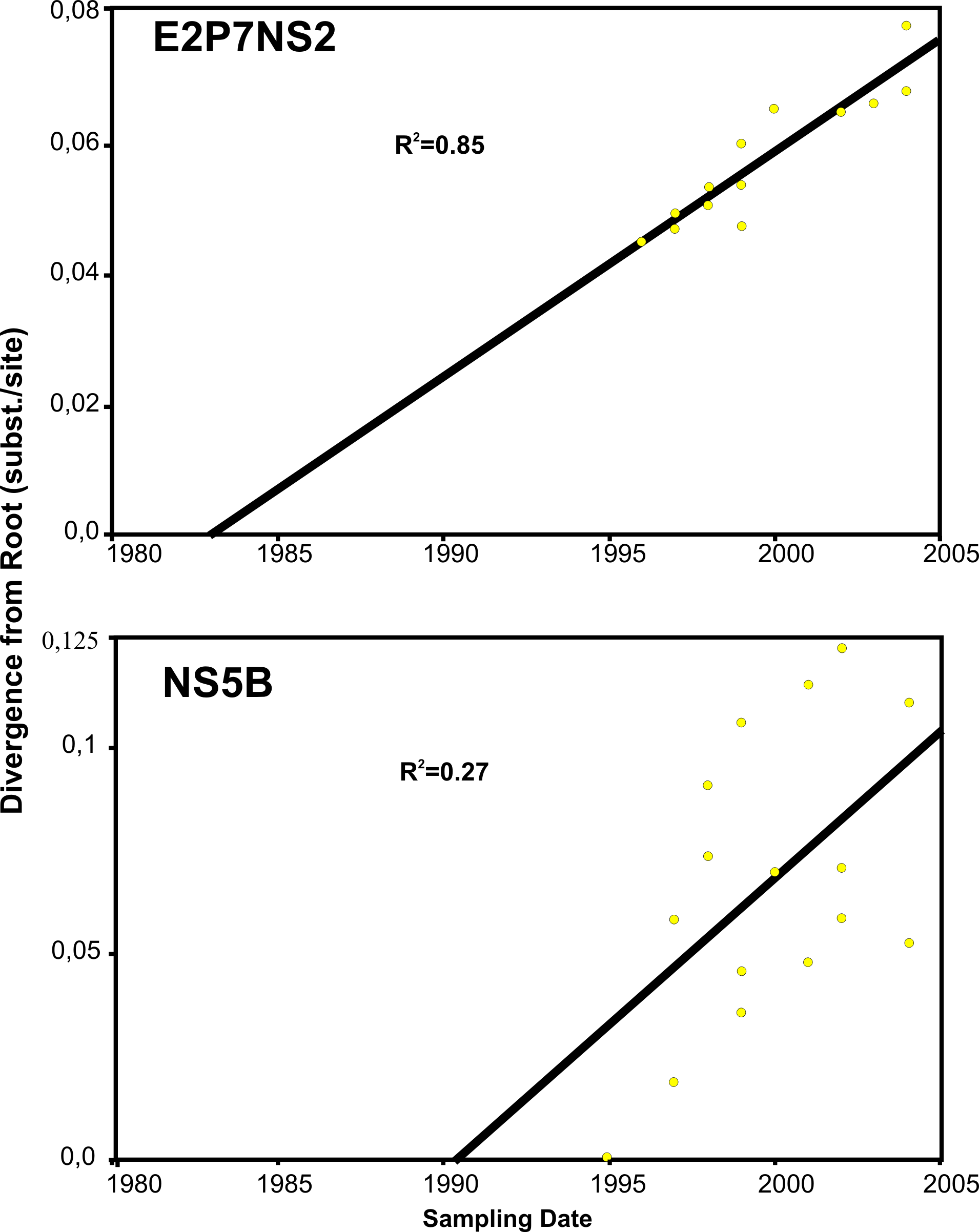

Supplement: Figure S1 — Regression of root-to-tip genetic distances against sampling date for the E2P7NS2 and NS5B regions in the model dataset (genotype 3a). The root has been chosen as the branch that maximizes the coefficient of determination (Pearson's r), under the assumption of a strict molecular clock. (0.41 MB TIF) [file pmed.1000198.s001.tif]

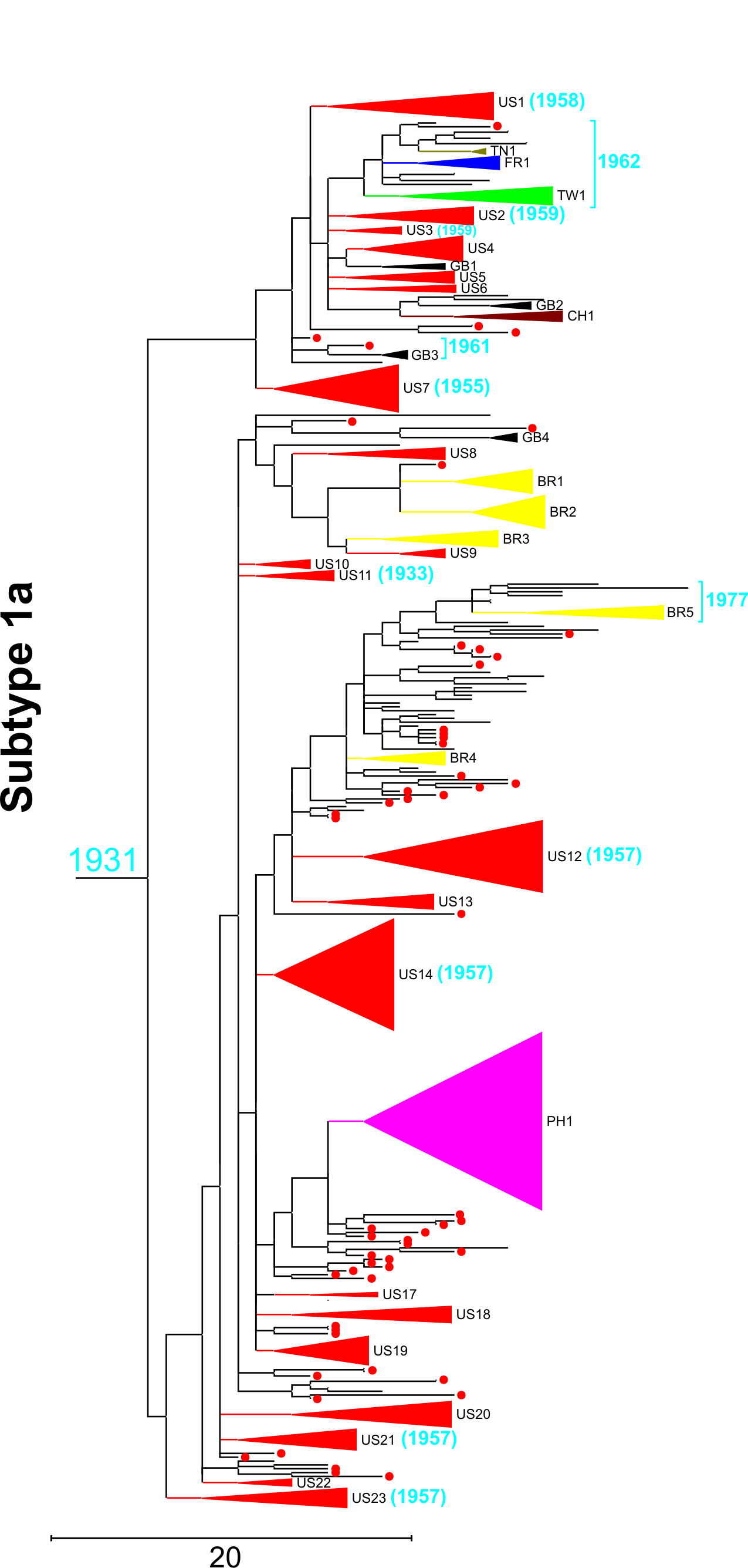

Supplement: Figure S2 — Phylogenetic trees of all partial NS5B sequences available for subtypes 1a (also presented as phylogeographic trees in Figure 2). This figure shows the phylogenetic trees annotated with dated nodes (median dates, blue numbers) and country-specific clusters (colored triangles). Each country-specific cluster is comprised of at least four taxa and contains at least 80% strains isolated from the specified country. Country codes are: ES (Spain), TN (Tunisia), US (United States of America), FR (France), GB (Great Britain), CH (Switzerland), BR (Brazil), PH (Philippines), TW (Taiwan), IE (Ireland), RU (Russia), IN (India), JP (Japan), CN (China), MN (Mongolia), VN (Vietnam). Red circles indicate dispersed strains isolated from the US. (0.40 MB TIF) [file pmed.1000198.s002.tif]

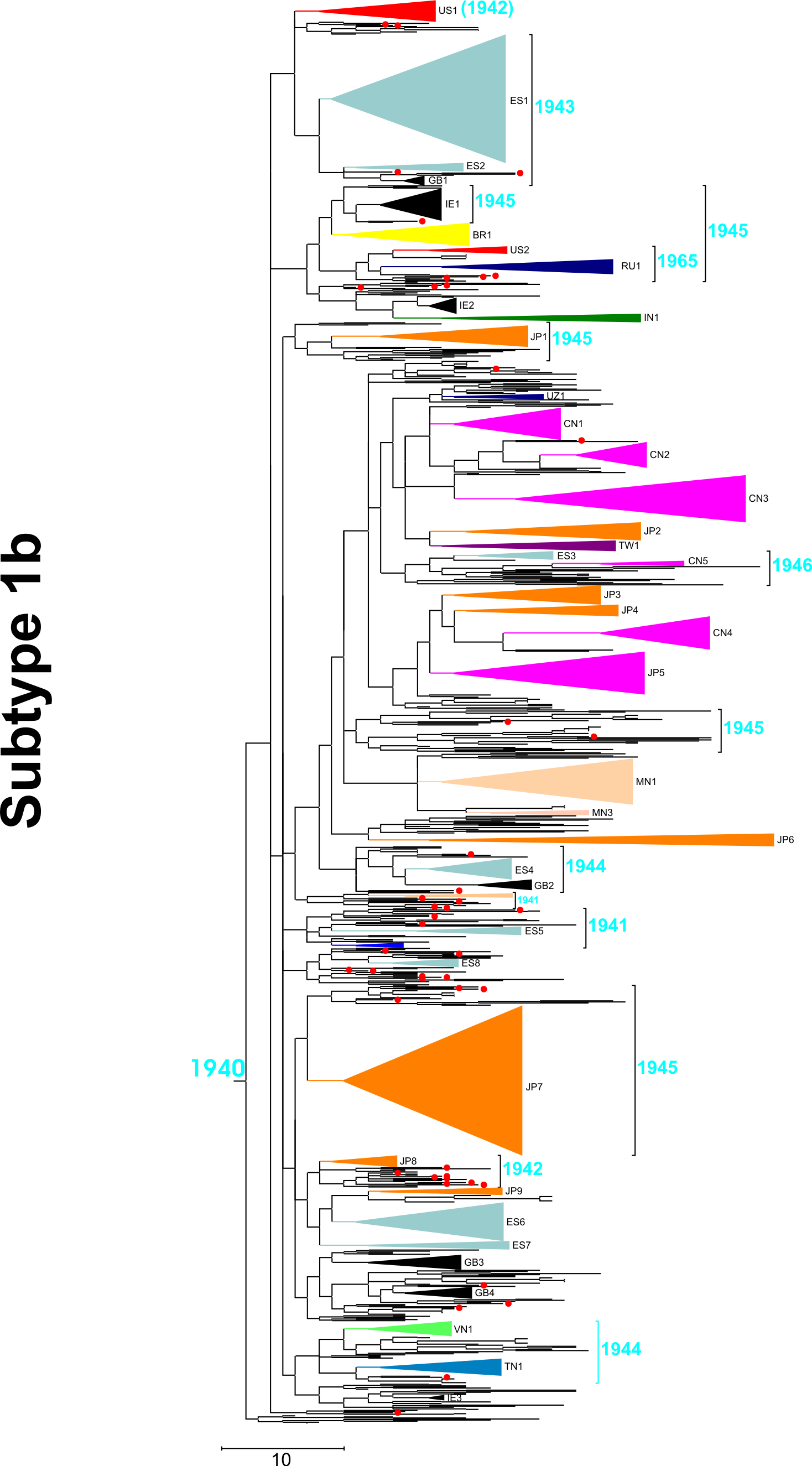

Supplement: Figure S3 — Phylogenetic trees of all partial NS5B sequences available for subtypes 1b (also presented as phylogeographic trees in Figure 2). This figure shows the phylogenetic trees annotated with dated nodes (median dates, blue numbers) and country-specific clusters (colored triangles). Each country-specific cluster is comprised of at least four taxa and contains at least 80% strains isolated from the specified country. Country codes are: ES (Spain), TN (Tunisia), US (United States of America), FR (France), GB (Great Britain), CH (Switzerland), BR (Brazil), PH (Philippines), TW (Taiwan), IE (Ireland), RU (Russia), IN (India), JP (Japan), CN (China), MN (Mongolia), VN (Vietnam). Red circles indicate dispersed strains isolated from the US. (0.42 MB TIF) [file pmed.1000198.s003.tif]
