# Supplementary material for: The Global Spread of Hepatitis C Virus 1a and 1b: A Phylodynamic and Phylogeographic Analysis
Source: PLoS Med. 2009 Dec 15;6(12):e1000198. doi: 10.1371/journal.pmed.1000198 (PMC2795363; doi:10.1371/journal.pmed.1000198)
Supplement: Table S1 — Epidemiological risk group distribution for each HCV subtype in the model dataset. Description of primers used in the experimental phase. (0.03 MB DOC) [file pmed.1000198.s004.doc]

|  | **Genotype 1a** | **Genotype 1b** | **Genotype 3a** | **Genotype 4a** |
| --- | --- | --- | --- | --- |
| Sampling Date Range  (Years) | 1994-2006 | 1994-2006 | 1995-2006 | 1995-2004 |
| **Risk Group**  (n) |  |  |  |  |
| Transfusion | 3 | 6 | 1 | 3 |
| Hemophiliac | 2 | 0 | 0 | 0 |
| IDU | 12 | 2 | 17 | 2 |
| Other* | 4 | 3 | 0 | 1 |
| Unknown | 3 | 16 | 6 | 16 |
| **Total** | 24 | 27 | 24 | 22 |

*Sexual partner of HCV carrier, hospitalization
