# Supplementary material for: The Global Spread of Hepatitis C Virus 1a and 1b: A Phylodynamic and Phylogeographic Analysis
Source: PLoS Med. 2009 Dec 15;6(12):e1000198. doi: 10.1371/journal.pmed.1000198 (PMC2795363; doi:10.1371/journal.pmed.1000198)
Supplement: Table S2 — Primers used to amplify the sequences forming the model dataset. For genotypes 1a, 1b, and 4a we have implemented a semi-nested approach for the E2P7NS2 region. (0.04 MB DOC) [file pmed.1000198.s005.doc]

| **Primer** | **Nucleotide sequence** | **Target region** | **Genotype** |
| --- | --- | --- | --- |
| **NS5B_SENSE** | YGAYACCCGITGYTTTGACTC | **NS5B** | **All** |
| **NS5B_ANTI** | RGTGTGICKIRCTGTYTCCC | **NS5B** | **All** |
|  |  |  |  |
| **GEN1_E_SENSE** | CCTGYYTRTGGATGATGYT | **E2P7NS2** | **1a/1b** |
| **GEN1_E_ANTI** | CASGTGATRABCTTGRTCTC | **E2P7NS2** | **1a/1b** |
| **GEN1_E_ANTI_IN** | CCTCVACYTGRTTYTTGTCC | **E2P7NS2** | **1a/1b** |
|  |  |  |  |
| **GEN3A_E_SENSE** | ATACACCTCCACCARAACATCG | **E2P7NS2** | **3a** |
| **GEN3A_E_ANTI** | GCTGSGCGTRTKCTGWGAT | **E2P7NS2** | **3a** |
|  |  |  |  |
| **GEN4A_E_SENSE** | CCAYCTCCAYCARAATATHGTGG | **E2P7NS2** | **4a** |
| **GEN4A_E_ANTI** | GTGTCMGCKCCCCASRYGATGA | **E2P7NS2** | **4a** |
| **GEN4A_E_ANTI_IN** | TACTTGCCATRRVYWAYCCCAC | **E2P7NS2** | **4a** |
