# Supplementary material for: The Global Spread of Hepatitis C Virus 1a and 1b: A Phylodynamic and Phylogeographic Analysis
Source: PLoS Med. 2009 Dec 15;6(12):e1000198. doi: 10.1371/journal.pmed.1000198 (PMC2795363; doi:10.1371/journal.pmed.1000198)
Supplement: Table S3 — Sequences of the global dataset, together with their spatiotemporal sampling information. (0.20 MB DOC) [file pmed.1000198.s006.doc]

a) Subtype 1a [1,2]

| **Sequence name** | **Country (ISO)** | **Sampling year** |
| --- | --- | --- |
| BID-V465 | US | 2007 |
| BID-V270 | CH | 2006 |
| BID-V698 | US | 2006 |
| BID-V696 | US | 2006 |
| BID-V437 | US | 2006 |
| BID-V409 | US | 2006 |
| BID-V407 | US | 2006 |
| BID-V406 | US | 2006 |
| BID-V404 | US | 2006 |
| BID-V401 | US | 2006 |
| BID-V399 | US | 2006 |
| BID-V398 | US | 2006 |
| BID-V396 | US | 2006 |
| BID-V391 | US | 2006 |
| BID-V390 | US | 2006 |
| BID-V362 | US | 2006 |
| BID-V359 | US | 2006 |
| BID-V358 | US | 2006 |
| BID-V337 | US | 2006 |
| BID-V336 | US | 2006 |
| BID-V1 | US | 2006 |
| BID-V457 | US | 2006 |
| BID-V450 | US | 2006 |
| BID-V445 | US | 2006 |
| BID-V101 | US | 2006 |
| BID-V269 | CH | 2005 |
| BID-V267 | CH | 2005 |
| BID-V246 | CH | 2005 |
| BID-V240 | CH | 2005 |
| BID-V239 | CH | 2005 |
| BID-V236 | CH | 2005 |
| BID-V388 | US | 2005 |
| BID-V90 | US | 2005 |
| BID-V86 | US | 2005 |
| BID-V104 | US | 2005 |
| BID-V35 | DE | 2004 |
| BID-V262 | CH | 2004 |
| BID-V414 | US | 2004 |
| BID-V357 | US | 2004 |
| BID-V73 | US | 2004 |
| BID-V68 | US | 2004 |
| BID-V54 | US | 2004 |
| BID-V105 | US | 2004 |
| BID-V29 | DE | 2003 |
| BID-V26 | DE | 2003 |
| BID-V257 | CH | 2003 |
| BID-V256 | CH | 2003 |
| BID-V413 | US | 2003 |
| BID-V411 | US | 2003 |
| BID-V356 | US | 2003 |
| BID-V335 | US | 2003 |
| BID-V434 | US | 2003 |
| BID-V93 | US | 2003 |
| BID-V91 | US | 2003 |
| BID-V221 | CH | 2002 |
| BID-V429 | US | 2002 |
| BID-V424 | US | 2002 |
| BID-V334 | US | 2002 |
| BID-V333 | US | 2002 |
| BID-V332 | US | 2002 |
| BID-V329 | US | 2002 |
| BID-V326 | US | 2002 |
| BID-V322 | US | 2002 |
| BID-V432 | US | 2002 |
| BID-V71 | US | 2002 |
| BID-V428 | US | 2001 |
| BID-V426 | US | 2001 |
| BID-V425 | US | 2001 |
| BID-V331 | US | 2001 |
| BID-V328 | US | 2001 |
| BID-V327 | US | 2001 |
| BID-V325 | US | 2001 |
| BID-V323 | US | 2001 |
| BID-V320 | US | 2001 |
| BID-V319 | US | 2001 |
| BID-V318 | US | 2001 |
| BID-V317 | US | 2001 |
| BID-V115 | US | 2001 |
| BID-V412 | US | 2000 |
| BID-V214 | US | 1996 |
| BID-V169 | US | 1996 |
| BID-V192 | US | 1992 |
| BID-V185 | US | 1991 |
| BID-V216 | US | 1990 |
| BID-V172 | US | 1990 |
| BID-V197 | US | 1989 |
| H-77 | _ | 1977 |

b) Subtype 1b [1]

| **Sequence name** | **Country (ISO)** | **Sampling Year** |
| --- | --- | --- |
| BID-V313 | CH | 2006 |
| BID-V311 | CH | 2006 |
| BID-V309 | CH | 2006 |
| BID-V289 | CH | 2006 |
| BID-V288 | CH | 2006 |
| BID-V385 | US | 2006 |
| BID-V375 | US | 2006 |
| BID-V374 | US | 2006 |
| BID-V371 | US | 2006 |
| BID-V369 | US | 2006 |
| BID-V367 | US | 2006 |
| BID-V366 | US | 2006 |
| BID-V365 | US | 2006 |
| BID-V363 | US | 2006 |
| BID-V449 | US | 2006 |
| BID-V447 | US | 2006 |
| BID-V146 | US | 2006 |
| BID-V308 | CH | 2005 |
| BID-V307 | CH | 2005 |
| BID-V301 | CH | 2005 |
| BID-V300 | CH | 2005 |
| BID-V285 | CH | 2005 |
| BID-V421 | US | 2005 |
| BID-V512 | US | 2005 |
| BID-V384 | US | 2005 |
| BID-V379 | US | 2005 |
| BID-V145 | US | 2005 |
| BID-V502 | DE | 2004 |
| BID-V306 | CH | 2004 |
| BID-V282 | CH | 2004 |
| BID-V281 | CH | 2004 |
| BID-V276 | CH | 2004 |
| BID-V159 | US | 2004 |
| BID-V156 | US | 2004 |
| BID-V150 | US | 2004 |
| BID-V148 | US | 2004 |
| BID-V147 | US | 2004 |
| BID-V142 | US | 2004 |
| BID-V504 | DE | 2003 |
| BID-V278 | CH | 2003 |
| BID-V275 | CH | 2003 |
| BID-V273 | CH | 2003 |
| BID-V354 | US | 2003 |
| BID-V347 | US | 2003 |
| BID-V158 | US | 2003 |
| BID-V157 | US | 2003 |
| BID-V155 | US | 2003 |
| BID-V152 | US | 2003 |
| BID-V297 | CH | 2002 |
| BID-V295 | CH | 2002 |
| BID-V294 | CH | 2002 |
| BID-V277 | CH | 2002 |
| BID-V420 | US | 2002 |
| BID-V419 | US | 2002 |
| BID-V353 | US | 2002 |
| BID-V352 | US | 2002 |
| BID-V350 | US | 2002 |
| BID-V341 | US | 2002 |
| BID-V164 | US | 2002 |
| BID-V163 | US | 2002 |
| BID-V160 | US | 2002 |
| BID-V151 | US | 2002 |
| BID-V296 | CH | 2001 |
| BID-V416 | US | 2001 |
| BID-V346 | US | 2001 |
| BID-V345 | US | 2001 |
| BID-V344 | US | 2001 |
| BID-V442 | US | 2001 |
| BID-V441 | US | 2001 |
| BID-V381 | US | 2001 |
| BID-V589 | RU | 1999 |
| BID-V132 | US | 1996 |
| BID-V130 | US | 1994 |
| BID-V139 | US | 1992 |
| BID-V136 | US | 1992 |
| BID-V128 | US | 1992 |
| BID-V127 | US | 1992 |
| BID-V125 | US | 1992 |
| BID-V124 | US | 1992 |
| BID-V121 | US | 1992 |
| BID-V122 | US | 1991 |
| BID-V141 | US | 1990 |
| BID-V134 | US | 1990 |
| BID-V131 | US | 1990 |
| BID-V133 | US | 1989 |

**References**

1. Henn MR, Kuntzen T, Young S, Kodira C, Koehrsen M, et al. (2007) Broad Institute Genome Sequencing Platform; Broad Institute Microbial Sequencing Center. Direct Submission.

2. Ogata N, Alter HJ, Miller RH, Purcell RH (1991) Nucleotide sequence and mutation rate of the H strain of hepatitis C virus. Proc Natl Acad Sci U S A 88: 3392-3396.
