# Supplementary material for: The Global Spread of Hepatitis C Virus 1a and 1b: A Phylodynamic and Phylogeographic Analysis
Source: PLoS Med. 2009 Dec 15;6(12):e1000198. doi: 10.1371/journal.pmed.1000198 (PMC2795363; doi:10.1371/journal.pmed.1000198)
Supplement: Table S4 — Country distribution of the global dataset. (0.02 MB PDF) [file pmed.1000198.s007.pdf]

Subtype 1a

Country	Frequency
rgentina	5
Canada	21
Switzerland	30
Brazil	96
Spain	10
Thailand	8
Great Britain	23
Vietnam	11
France-Martinique	24
Japan	3
Germany	9
China	2
India	2
Iran	2
Nepal	1
Philippines	161
Taiwan	11
Turkey	3
Greece	21
Not Determined	150
United States	399
Total	992

Subtype 1b

Country	Frequency
China	129
Japan	276
Mongolia	60
India	8
Nepal	1
Phillipines	3
Vietnam	13
Thailand	7
Taiwan	13
Singapore	4
Russia	22
Uzbekistan	31
France-Martinique	64
Belgium	2
Switzerland	24
Germany	7
Great Britain	40
Ireland	52
Spain	276
Brazil	33
Peru	1
Cameroon	1
Egypt	2
Tunisia	16
Madagarscar	9
Turkey	1
Kroatia	2
Greece	22
United States	89
Total	1,208
