# Supplementary material for: The Global Spread of Hepatitis C Virus 1a and 1b: A Phylodynamic and Phylogeographic Analysis
Source: PLoS Med. 2009 Dec 15;6(12):e1000198. doi: 10.1371/journal.pmed.1000198 (PMC2795363; doi:10.1371/journal.pmed.1000198)
Supplement: Table S5 — Model selection results for the subtype 1a global dataset. ln-likelihoods and log10 Bayes factors (BF) for each pair of models (model 1 = row versus model 2 = column). A log10 BF>5 (decibans) is substantial evidence and >10 is strong evidence for the support of model 1 over model 2. (0.05 MB DOC) [file pmed.1000198.s008.doc]

Subtype 1a

| **E2P7NS2** | ln P(data | model) | Relaxed - B.Skyline | Relaxed - Constant | Strict - B.Skyline | Strict - Constant | Strict - Logistic |
| --- | --- | --- | --- | --- | --- | --- |
| Relaxed - B.Skyline | -10244,127 | - | 63,07 | 4,302 | 81,999 | 12,259 |
| Relaxed - Constant | -10389,35 | -63,07 | - | -58,768 | 18,929 | -50,811 |
| Strict - B.Skyline | -10254,032 | -4,302 | 58,768 | - | 77,697 | 7,957 |
| Strict - Constant | -10432,936 | -81,999 | -18,929 | -77,697 | - | -69,74 |
| Strict - Logistic | -10272,354 | -12,259 | 50,811 | -7,957 | 69,74 | - |

| **NS5B** | ln P(data| model) | Relaxed - B.Skyline | Relaxed - Constant | Relaxed - Exponential | Relaxed - Logistic | Strict - B.Skyline | Strict - Constant | Strict - Exponential | Strict - Logistic |
| --- | --- | --- | --- | --- | --- | --- | --- | --- | --- |
| Relaxed - B.Skyline | -6434,086 | - | 67,696 | 3,108 | 13,547 | 12,181 | 79 | 8,876 | 16,336 |
| Relaxed - Constant | -6589,963 | -67,696 | - | -64,589 | -54,149 | -55,516 | 11,304 | -58,82 | -51,36 |
| Relaxed - Exponential | -6441,241 | -3,108 | 64,589 | - | 10,44 | 9,073 | 75,892 | 5,768 | 13,229 |
| Relaxed - Logistic | -6465,28 | -13,547 | 54,149 | -10,44 | - | -1,367 | 65,453 | -4,671 | 2,789 |
| Strict - B.Skyline | -6462,133 | -12,181 | 55,516 | -9,073 | 1,367 | - | 66,819 | -3,305 | 4,156 |
| Strict - Constant | -6615,99 | -79 | -11,304 | -75,892 | -65,453 | -66,819 | - | -70,124 | -62,664 |
| Strict - Exponential | -6454,524 | -8,876 | 58,82 | -5,768 | 4,671 | 3,305 | 70,124 | - | 7,46 |
| Strict - Logistic | -6471,702 | -16,336 | 51,36 | -13,229 | -2,789 | -4,156 | 62,664 | -7,46 | - |
