# Supplementary material for: The Global Spread of Hepatitis C Virus 1a and 1b: A Phylodynamic and Phylogeographic Analysis
Source: PLoS Med. 2009 Dec 15;6(12):e1000198. doi: 10.1371/journal.pmed.1000198 (PMC2795363; doi:10.1371/journal.pmed.1000198)
Supplement: Table S6 — Model selection results for the subtype 1b global dataset. ln-likelihoods and log10 Bayes factors (BF) for each pair of models (model 1 = row versus model 2 = column). A log10 BF>5 (decibans) is substantial evidence and >10 is strong evidence for the support of model 1 over model 2. (0.04 MB DOC) [file pmed.1000198.s009.doc]

Subtype 1b

| **E2P7NS2** | ln P(data | model) | Relaxed - B.Skyline | Relaxed - Constant | Relaxed - Logistic | Strict - B.Skyline | Strict - Constant | Strict - Exponential | Strict - Logistic |
| --- | --- | --- | --- | --- | --- | --- | --- | --- |
| Relaxed - B.Skyline | -14007,839 | - | 96,659 | 7,347 | 19,356 | 103,092 | 25,498 | 24,596 |
| Relaxed - Constant | -14230,405 | -96,659 | - | -89,312 | -77,302 | 6,434 | -71,161 | -72,063 |
| Relaxed - Logistic | -14024,756 | -7,347 | 89,312 | - | 12,01 | 95,746 | 18,151 | 17,249 |
| Strict - B.Skyline | -14052,409 | -19,356 | 77,302 | -12,01 | - | 83,736 | 6,141 | 5,24 |
| Strict - Constant | -14245,218 | -103,092 | -6,434 | -95,746 | -83,736 | - | -77,595 | -78,497 |
| Strict - Exponential | -14066,55 | -25,498 | 71,161 | -18,151 | -6,141 | 77,595 | - | -0,902 |
| Strict - Logistic | -14064,473 | -24,596 | 72,063 | -17,249 | -5,24 | 78,497 | 0,902 | - |

| **NS5B** | ln P(data | model) | Relaxed - B.Skyline | Relaxed - Constant | Strict - B.Skyline | Strict - Constant | Strict - Logistic |
| --- | --- | --- | --- | --- | --- | --- |
| Relaxed - B.Skyline | -7881,36 | - | 85,589 | 8,266 | 87,904 | 14,425 |
| Relaxed - Constant | -8078,436 | -85,589 | - | -77,324 | 2,314 | -71,164 |
| Strict - B.Skyline | -7900,392 | -8,266 | 77,324 | - | 79,638 | 6,159 |
| Strict - Constant | -8083,765 | -87,904 | -2,314 | -79,638 | - | -73,479 |
| Strict - Logistic | -7914,574 | -14,425 | 71,164 | -6,159 | 73,479 | - |
