# Supplementary material for: The Global Spread of Hepatitis C Virus 1a and 1b: A Phylodynamic and Phylogeographic Analysis
Source: PLoS Med. 2009 Dec 15;6(12):e1000198. doi: 10.1371/journal.pmed.1000198 (PMC2795363; doi:10.1371/journal.pmed.1000198)
Supplement: Table S7 — Comparison of the precision of different data combinations in estimating the tMRCA in the global dataset (95% higher posterior probability). It is easily shown that maximum precision is achieved when E2P7NS2's estimate of the tMRCA is applied as a prior on the tMRCA of NS5B. (0.03 MB DOC) [file pmed.1000198.s010.doc]

|  | **1a Prior** | **1a Combined** | **E2P7NS2** | **NS5B** |
| --- | --- | --- | --- | --- |
| **Date of MRCA**  **(95% HPD)** | 1906-1957 | 1858-1956 | 1818-1957 | 1805-1957 |
| **Number of sequences** | 108 | 99 | 101 | 108 |
| **Sampling range (Date)** | 1977-2007 | 1989-2007 | 1989-2007 | 1977-2007 |
|  |  |  |  |  |
|  | **1b Prior** | **1b Combined** | **E2P7NS2** | **NS5B** |
| **Date of MRCA**  **(95% HPD)** | 1921-1962 | 1886-1954 | 1904-1965 | 1805-1958 |
| **Number of sequences** | 108 | 100 | 105 | 108 |
| **Sampling range (Date)** | 1989-2006 | 1989-2006 | 1989-2006 | 1989-2006 |
