# Supplementary material for: The Global Spread of Hepatitis C Virus 1a and 1b: A Phylodynamic and Phylogeographic Analysis
Source: PLoS Med. 2009 Dec 15;6(12):e1000198. doi: 10.1371/journal.pmed.1000198 (PMC2795363; doi:10.1371/journal.pmed.1000198)
Supplement: Text S1 — Testing for random sampling and statistics about the time lag between subtype 1a and 1b epidemics. Analysis of Figures S1, S2, and S3. (0.04 MB DOC) [file pmed.1000198.s011.doc]

**1. Testing for random sampling**

In addition to visual inspection for random sampling, we also used a quantitative measurement of population sub-division, described by Slatkin and Maddison, in order to show that our sample of dated sequences does not differ significantly in terms of structure from a random sample taken from the global dataset. This analysis was performed by defining two groups of sequences in the global dataset: the dated sequences (1^st^ group) and the rest of the dataset (2^nd^ group). We then calculated the number of state changes between these two groups according to maximum parsimony. We subsequently compared this observed number of state changes with the distribution expected under the null hypothesis of no association between the phylogeny and the dated sub-sample. 500 replicate datasets were generated to obtain the null distribution; for each replicate, the 1^st^ group was defined as a set of sequences (equal in size to the number of dated sequences) that was randomly sampled from the whole dataset. The 2^nd^ group was defined as the remainder of the dataset.

For subtype 1a, the observed number of state changes fell within the distribution of 500 values obtained by simulating under the null hypothesis. On the other hand, for subtype 1b the observed number of state changes was significantly different from the distribution obtained from 500 null simulations. This departure indicates that the dated sequences are not equivalent in structure to a random sample of the same size. In order to elucidate the impact of this departure from random sampling on the reconstruction of epidemic history, we down-sampled the sample of dated sequences by removing 18 sequences contained in clusters of dated-sequences. This resulted in a new sample of dated sequences (1b sub-sample) that did not differ significantly from a random sample, as determined using the test outlined above. We subsequently repeated the phylodynamic analysis and compared the estimated epidemic history obtained using the 1b sub-sample with that obtained from original dated dataset. There was no significant difference of the median population growth curves (Kolmogorov Smirnov test; *p*=0.18), suggesting that this analysis is robust to the minor difference in sampling structure that we detected. Thus, in the final analysis we retained the whole dated dataset in order to have more statistical power.

**2. Time lag between subtype 1a and 1b epidemics**

Estimation of the temporal difference between the expansion of the subtype 1a and 1b epidemics is not straightforward. In order to describe the time lag between the two epidemics we consider the geometry of the epidemic expansion curves. There are at least 4 crucial points in these curves:

1. the tMRCA which is when the curve begins; this point indicates the common ancestor of the sample and is often used to represent the temporal origin of an epidemic
2. the start of the exponential growth period; this point indicates when the epidemic started to grow rapidly
3. the middle of the exponential growth period; in logistic growth, this describes the point at which growth rate is maximal
4. the end of the exponential growth (beginning of the plateau); this point indicates when growth declines to zero and population prevalence stabilizes.

To determine the time lag between epidemics it is crucial to select the most appropriate time point. The tMRCA is not a valid time point to use because it describes only the date of common ancestry, not when growth began. If there is a period of latent or slow transmission prior to an epidemic (which differs in length among subtypes) then the tMRCA will be misleading as a measure of time lag. The end of the exponential growth period is also uninformative in this respect.

In order to describe the time lag between the two epidemics we use the start and the middle point of the exponential growth period. Estimation of the posterior distribution of both of these points is not straightforward. To estimate these values, we simulated 1000 replicate exponential growth curves for each subtype, with each replicate having start and midpoint times sampled from the following ranges (values represent ‘years before 2007’):

1. Start of the exponential growth period:

subtype1a: 95% range = [37-65.8], median = 43.2

subtype1b: 95% range = [52.6-60.8], median = 60.8

1. Middle of the exponential growth period:

subtype 1a: 95% range = [28.78-51.4], median = 37

subtype 1b: 95% range = [40.5-59.5], median = 59.5

These distributions are constructed using the 95 % higher posterior density of the respective Bayesian skylines. For subtype 1b the upper 95% credibility interval of the date of these points has been constrained on the median since the upper 50% of the higher posterior density is presented to be concentrated on the same time point. These constrained distributions, as previously stated, are the most conservative to be assumed in regards with the time-lag null hypothesis: these are the smaller values that can be assumed for the upper 50% of the points of interest for subtype 1b and, thus, they are favourable for smaller time lags between 1a and 1b.

For each simulated replicate, we calculated the median time lag between the two subtypes, which was 16.1 (95% C.I.: 15.3-16.8) and 16.4 (95% C.I.: 15.8-17.1) years for the start and the midpoint of the exponential phase, respectively.

Supplementary Figure S1. Regression of root-to-tip genetic distances against sampling date for the E2P7NS2 and NS5B regions in the model dataset (genotype 3a). The root has been chosen as the branch that maximizes the coefficient of determination (Pearson’s r), under the assumption of a strict molecular clock.

Supplementary Figures S2 and S3. The phylogenetic trees of all partial NS5B sequences available for subtypes 1a and 1b (also presented as phylogeographic trees in Figure 2). This figure shows the phylogenetic trees annotated with dated nodes (median dates, blue numbers) and country-specific clusters (colored triangles). Each country-specific cluster is comprised of at least 4 taxa and contains at least 80% strains isolated from the specified country. Country codes are: ES (Spain), TN (Tunisia), US (United States of America), FR (France), GB (Great Britain), CH (Switzerland), BR (Brazil), PH (Philippines), TW (Taiwan), IE (Ireland), RU (Russia), IN (India), JP (Japan), CN (China), MN (Mongolia), VN (Vietnam). Red circles indicate dispersed strains isolated from the US.
